# Targeting Cancer with Redox Catalysis: Manganese Porphyrins and Ascorbate Synergistically Induce Selective Oxidative Stress and Necrotic Cell Death

**DOI:** 10.3390/cancers17233736

**Published:** 2025-11-22

**Authors:** Michał Rąpała, Maciej Pudełek, Sławomir Lasota, Sylwia Noga, Jarosław Czyż, Janusz M. Dąbrowski, Zbigniew Madeja

**Affiliations:** 1 Department of Cell Biology, Faculty of Biochemistry, Biophysics and Biotechnology, Jagiellonian University in Kraków, ul. Gronostajowa 7, 30-387 Kraków, Poland; maciej.pudelek@uj.edu.pl (M.P.); slawomir.lasota@uj.edu.pl (S.L.); sylwia.noga@uj.edu.pl (S.N.); jarek.czyz@uj.edu.pl (J.C.); 2 Doctoral School of Exact and Natural Sciences, Jagiellonian University in Kraków, ul. prof. S. Łojasiewicza 11, 30-348 Kraków, Poland; 3 Faculty of Chemistry, Jagiellonian University in Kraków, 30-387 Kraków, Poland; janusz.dabrowski@uj.edu.pl

**Keywords:** manganese porphyrins, vitamin C, redox catalysis, reactive oxygen species, cancer therapy

## Abstract

Background: The combination of manganese porphyrins (MnPs) and ascorbate (ASC) represents a promising redox-based therapeutic approach for selectively targeting cancer cells. We investigated the cytotoxic effects of two structurally distinct MnPs (MnTPPS and MnF_2_BMet) with differing lipophilicity and potential membrane permeability in combination with ASC. Methods: Cancer cell lines (MCF-7, PANC-1, U87, T98G, AT-2) and normal human dermal fibroblasts (HDFs) were treated with MnTPPS and MnF_2_BMet in the absence or presence of ASC. Viability, migration potential, and intracellular oxidative stress were assessed using single-cell methods. Results: MnPs alone exhibited no intrinsic cytostatic or cytotoxic activity, as confirmed by proliferation, viability, and motility assays. When combined with ASC, both MnTPPS and MnF_2_BMet significantly enhanced ASC-induced oxidative stress, leading to lipid peroxidation, glutathione depletion, mitochondrial dysfunction, and cell membrane disruption. Time-lapse microscopy revealed rapid necrotic cell death under co-treatment. Catalase fully abolished cytotoxicity, indicating the essential role of hydrogen peroxide. In contrast, dehydroascorbate (DHA), which increases intracellular ASC levels, did not induce the same toxicity, suggesting that extracellular ROS generation contributes predominantly to the observed effects. Normal fibroblasts were minimally affected, supporting the MnPs–ASC system’s selectivity toward cancer cells. Conclusions: MnTPPS and MnF_2_BMet enhance extracellular oxidation of ascorbate and subsequent ROS production, leading to selective oxidative-stress-mediated cancer cell death. This study supports the potential of MnPs–ASC redox catalysis as a complementary oxidative-stress-based anticancer strategy and highlights the need for further mechanistic and structure–activity investigations.

## Simple Summary

Cancer cells are generally more vulnerable to oxidative stress than normal cells. Compounds capable of modulating reactive oxygen species (ROS) levels may therefore preferentially damage malignant cells. In this study, we investigated manganese porphyrins, a group of redox-active molecules that can interact with vitamin C (ascorbate) to promote oxidative reactions. Our goal was to clarify how these compounds influence cancer cell viability and whether they enhance oxidative-stress-mediated cell death. We found that combining manganese porphyrins with ascorbate increases ROS formation in the extracellular environment, which contributes to membrane disruption and loss of cell integrity. These findings help explain how manganese porphyrins can potentiate redox-based anticancer approaches and support the development of selective oxidative-stress-inducing therapies.

## 1. Introduction

Porphyrins are polycyclic aromatic compounds consisting of four pyrrole rings interconnected by methine bridges, forming an extended conjugated π-electron system with 18 delocalized electrons. Their ability to coordinate metal ions within the macrocyclic cavity determines their unique catalytic properties. These molecules are fundamental components of numerous biologically significant proteins, playing key roles in essential processes such as oxygen transport, electron transfer, and enzymatic catalysis. A prominent example is heme, which serves as a prosthetic group in hemoglobin and cytochromes, where the coordinated iron ion (Fe^2+^) undergoes cyclic redox reactions, facilitating electron transport in processes such as cellular respiration [1]. Beyond their biological significance, porphyrins exhibit distinctive photochemical and catalytic properties, making them attractive for biomedical applications, particularly in oncology. Their roles as photosensitizers in photodynamic therapy (PDT) and as contrast agents for fluorescence and photoacoustic imaging are well established [2]. Upon light absorption, porphyrins undergo a transition to triplet excited states, subsequently generating reactive oxygen species (ROS), which trigger oxidative stress, leading to apoptosis or necrosis of targeted cells. Furthermore, photogenerated ROS contribute to tumor vasculature disruption, trigger inflammatory responses, enhance the expression of heat shock proteins, and promote immune cell infiltration, thereby supporting the development of long-term immunological memory [3,4]. A key advantage of PDT lies in its spatial selectivity, as ROS production occurs exclusively in the illuminated region, minimizing systemic side effects [5,6]. However, the clinical use of porphyrin-based photosensitizers in PDT is limited by poor light penetration into deep tissues, restricting its efficacy mainly to superficial tumors. To address this limitation, alternative strategies have been explored, including chemical modulation, such as structural modifications involving the reduction of double bonds in pyrrole rings, attachment of substituents to enhance long-wavelength absorption and biocompatibility, or the addition of adjuvants to improve therapeutic efficacy [7]. Among potential adjuvants, ascorbate (vitamin C) has attracted considerable attention for its ability to enhance the therapeutic efficacy of porphyrin-based systems, particularly when light activation is not feasible [8].

L-ascorbic acid (AA) is a key regulator of lysyl and prolyl hydroxylase activity, making it indispensable for collagen biosynthesis [9]. It plays a fundamental role in maintaining connective tissue homeostasis; however, due to a mutation in the gene encoding L-gulono-γ-lactone oxidase, humans lack the ability to synthesize it endogenously, so it must be acquired through dietary intake. Under physiological conditions, the plasma concentration of AA is approximately 90 µM, with a maximum level of about 220 µM achievable through oral supplementation. At these concentrations, AA primarily functions as an antioxidant, scavenging ROS to protect cells from oxidative stress and prevent the degradation of subcellular structures [10]. However, at pharmacological concentrations, AA exerts a distinct prooxidant effect, which has been linked to its potential anticancer activity. Intravenous administration can elevate plasma ASC levels to approximately 0.5 mM without causing severe adverse effects [11]. Transient plasma concentrations as high as 20 mM can be achieved, leading to a disruption of cellular redox homeostasis. This oxidative imbalance contributes to glutathione oxidation and depletion of intracellular antioxidant reserves, as well as the redox cycling of iron ions, which often accumulate in cancer cells due to ferritin overexpression. These processes facilitate the generation of hydroxyl radicals via the Fenton and Haber–Weiss reactions, ultimately contributing to cytotoxic effects [12].

The cytotoxic effect of pharmacological ascorbate appears to be selective for cancer cells, which often display weakened antioxidant defense systems and increased susceptibility to ferroptosis resulting from metal-ion accumulation [12,13,14,15]. Additionally, certain tissues, particularly tumors, can locally accumulate higher concentrations of ASC, further enhancing its cytotoxic potential [16,17,18,19]. Cellular uptake of vitamin C occurs primarily through sodium-dependent vitamin C transporters (SVCT-1 in intestinal epithelial cells and SVCT-2 in other tissues) [15,16], as well as glucose transporters (GLUT). GLUTs mediate the uptake of DHA, the oxidized form of ascorbate, which is structurally similar to glucose. After DHA enters cells through GLUTs, it is subsequently reduced intracellularly to ASC. This mechanism is particularly relevant in cancer cells, which exhibit increased glucose uptake and GLUT overexpression, enabling preferential accumulation of ASC in tumor tissue [10]. Some studies also suggest that a fraction of ASC may directly diffuse through lipid membranes, although this is considered a secondary uptake mechanism [11]. Both in vitro and clinical studies indicate that pharmacological doses of vitamin C can exert selective cytotoxic effects on cancer cells, presenting a promising adjunctive strategy in oncology [20]. An emerging therapeutic concept involves the combination of ASC and manganese porphyrins as a complementary approach to conventional porphyrin-based photodynamic therapy (PDT), potentially enhancing therapeutic efficacy.

Manganese porphyrins (MnPs) have been explored as redox-active compounds with potential roles in radio- and chemosensitization, and some analogs have also shown radioprotective properties in normal tissues [21]. This dual function, combined with their superoxide dismutase (SOD)-mimicking catalytic activity, makes them particularly valuable in oncology, as they can enhance the effectiveness of radiotherapy and chemotherapy while mitigating radiation-induced damage to normal cells. Several MnPs have been developed that catalyze the oxidation of ASC and thiols, generating ROS. The redox potential (E_1_/_2_) of the Mn^3+^/Mn^2+^ couple plays a crucial role in determining the ability of Mn^3+^ to participate in redox cycling with ASC [22]. When the half-cell reduction potential (E_1_/_2_) of MnPs is well-matched with the redox potential of the Asc^•−^/AscH^−^ pair, ROS production via redox cycling is significantly enhanced [23]. Several MnPs have already been tested in combination with ASC on cancer cells, yielding promising therapeutic outcomes [21,22,23,24].

These findings prompted us to investigate the biological activity of two manganese porphyrins, MnTPPS and MnF_2_BMet, obtained following a general synthetic route previously described for related porphyrins prepared for other applications. These compounds are structurally related to porphyrin-based photosensitizers used in PDT, but their design allows them to undergo redox reactions without light activation [7,25,26,27]. In particular, we aimed to assess the synergy between the cytotoxic effects of these MnPs and ASC. Furthermore, we explored the molecular mechanisms underlying this cytotoxicity, with particular attention to the role of hydrogen peroxide (H_2_O_2_) in mediating these effects. To achieve this, we employed an experimental model integrating analyses of glutathione (GSH) depletion, lipid peroxidation, and direct H_2_O_2_ quantification in a panel of normal and cancer cell lines treated with MnPs/ASC in vitro.

## 2. Materials and Methods

### 2.1. Cell Lines

Human MCF-7 (ATCC^®^ HTB-22™) (Manassas, VA, USA), PANC-1 (ATCC^®^ CRL-1469™) (Manassas, VA, USA), cell lines were purchased from ATCC (Manassas, VA, USA). Rat prostate adenocarcinoma AT-2 (RRID:CVCL_L303) cells were kindly provided by Prof. Mustafa B. A. Djamgoz (Imperial College London, UK). Cells were cultured in RPMI-1640 medium (Sigma-Aldrich Co. LLC, (St. Louis, MO, USA) under standard conditions (37 °C, 5% CO_2_). The AT-2 cell line was established as described previously [28]. Glioblastoma cell lines U87 (ATCC^®^ nr HTB-14™) (Manassas, VA, USA) and T98G (ATCC^®^ nr CRL-1690™) (Manassas, VA, USA), along with normal human dermal fibroblasts (HDF, ATCC^®^ nr PCS-201-012 ^™^) (Manassas, VA, USA), were maintained under identical conditions in DMEM-high glucose medium (Sigma-Aldrich Co. LLC, (St. Louis, MO, USA). Culture media were supplemented with 10% heat-inactivated fetal bovine serum (FBS; GibcoTM, Paisley, UK) and 1% Antibiotic-Antimycotic Solution (Sigma-Aldrich Co. LLC, (St. Louis, MO, USA; 100 U/mL penicillin, 0.1 mg/mL streptomycin, 0.25 µg/mL amphotericin B). Cells were harvested using TrypLE™ (GibcoTM, Paisley, UK), counted using a Z2 particle counter (Beckman Coulter (Brea, CA, USA)), and seeded into multi-well tissue culture plates (Falcon^®^ Corning Inc. (Durham, NC, USA). All cell lines were maintained under standard culture conditions and routinely tested for mycoplasma contamination.

### 2.2. Manganese Porphyrins

Mn(III) tetrakis(4-benzoic acid) porphyrin chloride (MnTBAP) was purchased from Sigma-Aldrich Co. LLC, (St. Louis, MO, USA. The manganese(III) porphyrins were prepared following a general multistep procedure commonly used for related metalloporphyrins, involving condensation via the nitrobenzene method, chlorosulfonation, and subsequent manganese(III) complexation [29]. Solid manganese porphyrins (MnPs) were stored in light-protected vials at −20 °C. When dissolved in DMSO (Gibco TM, Paisley, UK), solutions were stored at 4 °C in light-protected vials. MnPs were added to cell cultures for 24 h, after which the medium was removed, and cells were washed with PBS (Gibco TM, Paisley, UK) to eliminate unbound porphyrins. The final DMSO concentration in the medium was 0.1% for 5 µM porphyrins and 1% for 50 µM porphyrins. MnTPPS and MnF_2_BMet were used as fully dissolved molecular species, which prevents colloidal aggregation; therefore, parameters such as hydrodynamic size or ζ-potential are not applicable to these systems. Control samples received the same DMSO concentration without porphyrins.

### 2.3. Optical Properties

Electronic absorption spectra of MnTPPS and MnF_2_BMet were recorded in PBS using quartz cuvettes with a 1 cm path length. Measurements were performed with a UV-3600 Shimadzu spectrophotometer (Shimadzu Corporation, Kyoto, Japan), covering the 300–800 nm wavelength range. Fluorescence spectra were acquired in the 500–750 nm range following excitation at the respective Soret bands. Measurements were conducted using an RF-6000 Shimadzu Spectrophotometer (Shimadzu Corporation, Kyoto, Japan). Samples were initially adjusted to an absorbance of 0.2 at the Soret band and subsequently diluted 100-fold for fluorescence analysis.

### 2.4. Cytotoxicity Assay

In all experiments, a sequential treatment approach was applied: cells were incubated with MnPs for 24 h, after which unbound porphyrins were removed by washing with PBS (Sigma-Aldrich Co. LLC, (St. Louis, MO, USA)). ASC (Sigma-Aldrich Co. LLC, (St. Louis, MO, USA))was added in fresh medium. The ascorbate solution (Sigma-Aldrich Co. LLC, (St. Louis, MO, USA)) was freshly prepared immediately before use. Cells were seeded into 24-well plates at a density of 10,000 cells/well. After 24 h of incubation, cell viability was assessed using the Trypan Blue ((Sigma-Aldrich Co. LLC, (St. Louis, MO, USA) exclusion assay. The cytotoxicity of MnPs was evaluated after the same incubation period, with the same procedure but without adding ASC. The experiments were conducted in three biological replicates.

For proliferation analysis, cells were seeded in 12-well plates at a density of 10,000 cells per well and then incubated for 24 h with MnPs. Subsequently, unbound porphyrins were removed, and fresh culture medium containing ASC was added. Cells were harvested after 96 h of incubation with ASC. Cells were counted using a Z2 particle counter (Beckman Coulter (Brea, CA, USA)). The experiments were performed in three biological replicates. Experiments with extracellular catalase were conducted using catalase from bovine liver (Sigma-Aldrich Co. LLC, (St. Louis, MO, USA). Catalase was dissolved in PBS and added to the culture medium at a final concentration of 200–500 U/mL.

### 2.5. Kinetics of Propidium Iodide Uptake

Propidium iodide (PI) (Sigma-Aldrich Co. LLC, (St. Louis, MO, USA) uptake kinetics were monitored using time-lapse videomicroscopy on 24-well plates (Falcon^®^ Corning Inc. (Durham, NC, USA). MCF-7 cells were seeded at a density of 10,000 cells/well and incubated with 5 µM MnTPPS or 5 µM MnF_2_BMet for 24 h. After removing unbound porphyrins, fresh medium containing 0.5 or 1 mM ASC was added, along with propidium iodide. Cell viability was monitored for 12 h at 5 min intervals using a Leica DMI6000B imaging system equipped with integrated modulation contrast (IMC; Hoffman contrast) (GmbH, Wetzlar, Germany), a fluorescence module, and environmental control for temperature (37 °C) and CO_2_ (5%). Propidium iodide fluorescence intensity was measured over time in specific cells (*n* = 30) collected from three biological replicates. Cell imaging began 30 min after ASC addition.

### 2.6. Detection of Caspase-3/7 Activation

Caspase-3 and caspase-7 activity induced by MnPs-ASC was assessed using CellEvent™ Caspase-3/7 Detection Reagents Green (Gibco TM, Paisley, UK). Cells were seeded and imaged as described above in section “Kinetics of propidium iodide uptake”. Caspase-3/7 Detection Reagents were added 30 min prior to ASC treatment, and cells were imaged in the green fluorescence channel. The experiment was performed in three biological replicates.

### 2.7. Cell Migration Assay

MCF-7 cell migration was analyzed using 24-well plates (Falcon^®^ Corning Inc. (Durham, NC, USA). Cells were seeded at a density of 10,000 cells/well and incubated with 5 µM MnTPPS or 5 µM MnF_2_BMet for 24 h. After removing unbound porphyrins, fresh medium containing ascorbate was added. Cell movement was recorded for 8 h at 5 min intervals using a Leica DMI6000B imaging system equipped with integrated modulation contrast (IMC; Hoffman contrast), CO_2_ (5%), and temperature (37 °C) monitoring. A total of 60 cells (*n* = 60) from three biological replicates were used for the analysis. Cell migration trajectories were manually tracked using Hiro v.1.0.0.4 software (developed by W. Czapla), and the speed of movement (µm/min) was calculated [30].

### 2.8. Fluorescence Microscopy

For fluorescence microscopy studies, MCF-7 cells were seeded into 12-well plates on UVC-sterilized coverslips at a density of 10,000 cells/well. Cells were incubated for 24 h with 5 µM MnPs, washed with PBS, and subsequently incubated with 0.5 mM ASC for 6 h. Cells were then fixed with 3.7% formaldehyde (Pol-Aura (Pol-Aura Sp. z o.o., Zawroty, Poland) and permeabilized with 0.1% Triton X-100 (Sigma-Aldrich Co. LLC, (St. Louis, MO, USA). Non-specific binding sites were blocked with 2% BSA (Invitrogen™ (Waltham, MA, USA). Actin cytoskeleton visualization was performed using AlexaFluor546-conjugated phalloidin (1:80), and nuclear staining was performed with Hoechst 33,258 (Sigma-Aldrich Co. LLC, (St. Louis, MO, USA), 1–2 µg/mL). After 45 min of incubation, samples were mounted in Moviol 4–88 mounting medium. Images were acquired using a Leica Stellaris 5 confocal microscope (Leica-microsystems (Ernst-Leitz-Straße, Wetzlar, DE)) [31].

### 2.9. Quantification of GSH, Lipid Peroxidation, and Mitochondrial Membrane Potential

MCF-7 cells were seeded at 10,000 cells/well in 24-well plates (Eppendorf AG (Hamburg, Germany)). Cells were incubated with 5 µM MnTPPS or 5 µM MnF_2_BMet for 24 h, washed with PBS, and incubated in phenol red-free RPMI 1640 medium. GSH levels were quantified using the ThiolTracker™ Violet assay (Invitrogen™(Waltham, MA, USA).) after 2 h of incubation with 0.5 mM ASC. Lipid peroxidation was assessed using the Image-iT™ Lipid Peroxidation Kit (Invitrogen™ (Waltham, MA, USA).) according to the manufacturer’s protocol. Mitochondrial membrane potential was analyzed using the JC-1 (Sigma-Aldrich Co. LLC, (St. Louis, MO, USA) fluorescence probe in glass-bottom culture dishes (Thermo Fisher™ (Waltham, MA, USA)). MCF-7 cells were preloaded with 20 µM MnF_2_BMet for 24 h, washed with PBS, and incubated in phenol red-free RPMI 1640 (Sigma-Aldrich Co. LLC, (St. Louis, MO, USA) [32]. The green-to-red fluorescence ratio was measured 2 h after the addition of 0.5 mM ASC. All imaging was performed using a Leica Stellaris 5 microscope (Leica-microsystems (Ernst-Leitz-Straße, Wetzlar, DE) equipped with a CO_2_ chamber (5%) and temperature control (37 °C). All microscopy analyses were performed by examining 50 cells from three biological replicates.

### 2.10. Quantification of Intracellular H_2_O_2_

Intracellular hydrogen peroxide levels were measured using the HyPer7 fluorescent probe [33]. MCF-7 cells were transfected with pCS2 + HyPer7-NES plasmid (kindly provided by Vsevolod Belousov; Addgene plasmid #136467) using Lipofectamine 3000 (Invitrogen™ (Waltham, MA, USA).), according to the manufacturer’s protocol. Twenty-four hours later, 5 µM MnF_2_BMet was added, and the cells were incubated in its presence for the next 24 h. Imaging was performed in glass-bottom culture dishes (Ibidi (GmbH, Gräfelfing, Germany)) using a Leica Stellaris 5 confocal microscope with CO_2_ (5%) and temperature control (37 °C). Images were collected at 30 s intervals over 6 min. After the initial 90 s of imaging, ASC was added to achieve a final concentration of 0.1 mM. Cells were imaged using 405 nm and 488 nm laser excitation, and emission was measured in the green fluorescence channel. A 405 nm laser (50 mW) was operated at 0.735% of its intensity, and a 488 nm laser (20 mW) was operated at 0.118% of its intensity. The combined pixel dwell time during image acquisition was 3.1625 µs. Fluorescence analysis was performed using a custom ImageJ macro (ImageJ 1.54j.), which generated 488/405 nm ratiometric images by pixel-by-pixel division and applied threshold-based masks to automatically detect individual cells. For each time point, the average ratio within each cell was calculated.

### 2.11. Quantification of Intracellular Ascorbate Levels

Intracellular ASC levels were measured using the Ascorbic Acid Assay Kit II (Sigma-Aldrich Co. LLC, (St. Louis, MO, USA). MCF-7 cells (1 × 10^6^) were seeded in culture dishes and treated with either 0.5 or 2 mM ASC or 0.5 or 2 mM DHA in PBS for 90 min. Then, the cells were washed twice with PBS, lysed, and centrifuged to remove cellular debris. Proteins were eliminated from the supernatant with a 10 kDa MWCO spin filter (Sigma-Aldrich Co. LLC, (St. Louis, MO, USA), and the reducing activity of each sample was measured at 595 nm. For each sample, a control containing ASC oxidase was included to distinguish between the oxidized and reduced forms of ASC. All samples were prepared in triplicate to ensure statistical accuracy.

### 2.12. Statistical Analysis

The statistical significance of variances in cell migration speed, cell viability, and fluorescence assay results was evaluated using Student’s t-test. All statistical analyses were conducted using STATISTICA 13.3, with the threshold for statistical significance set at *p* < 0.05.

## 3. Results

### 3.1. Optical Properties of Manganese Porphyrins

**Figure 1:**
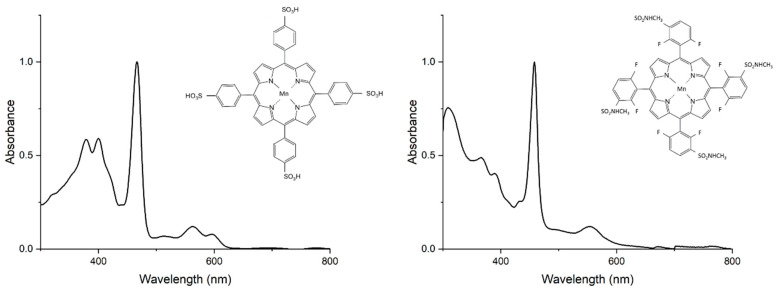
Chemical structures and electronic absorption spectra of 5,10,15,20-Tetrakis(4-sulfonylphenyl)porphyrin manganese(III) acetate (MnTPPS) and 5,10,15,20-Tetrakis [2,6-difluoro-5(*n*-methylsulfamoyl)phenyl]porphyrin manganese(III) (MnF_2_BMet) recorded in PBS or PBS with 0.5% DMSO at room temperature.

Figure 1 presents the structures of the two studied manganese(III) porphyrins: 5,10,15,20-Tetrakis(4-sulfonylphenyl)porphyrin manganese(III) acetate (MnTPPS) and 5,10,15,20-Tetrakis [2,6-difluoro-5(*n*-methylsulfamoyl)phenyl]porphyrin manganese(III) (MnF_2_BMet) porphyrins, which differ in their *meso*- or *para*-substituents (sulfonyl, sulfonamide) and *ortho*-halogen substituents. These structural variations may influence their stability and lipophilicity, which, in turn, can potentially affect their cellular distribution, localization, and biological activity. The electronic absorption spectra of these metal complexes are demonstrated in Figure 1. Emission spectra were recorded; fluorescence was negligible under our conditions, which is consistent with strong excited-state quenching in Mn(III) porphyrins.

The electronic absorption spectra showing characteristic Soret and Q bands are consistent with Mn(III) porphyrins of high purity; fluorescence quenching is expected for Mn(III) complexes. Both complexes exhibit a strong Soret band in the 400–500 nm region and additional charge-transfer features that extend into the 300–400 nm region. These additional bands arise from charge-transfer (CT) transitions, which are especially pronounced in high-spin manganese(III) porphyrins. In manganese(III) complexes, these transitions include metal-to-ligand charge transfer (MLCT), involving electron density from manganese d orbitals to porphyrin π* orbitals, and ligand-to-metal charge transfer (LMCT), where electrons transfer from porphyrin π orbitals to manganese d orbitals. These features define hyper-d-type spectra, characteristic of high-spin manganese(III) porphyrins, and explain their electronic absorption properties shown in Figure 1.

### 3.2. Selective Cytotoxicity of the MnPs-ASC System Against Cancer Cells: A Screening Approach

To evaluate the cancer cell specificity of the cytotoxic effects exerted by the MnPs/ASC system, we conducted a screening study across a range of selected human glioblastoma, prostate, and pancreatic cancer cell lines (MCF-7, PANC-1, U87, and T98G) and compared their responses to those of rat prostate cancer cells (AT-2) and normal human dermal fibroblasts (HDFs).

**Figure 2:**
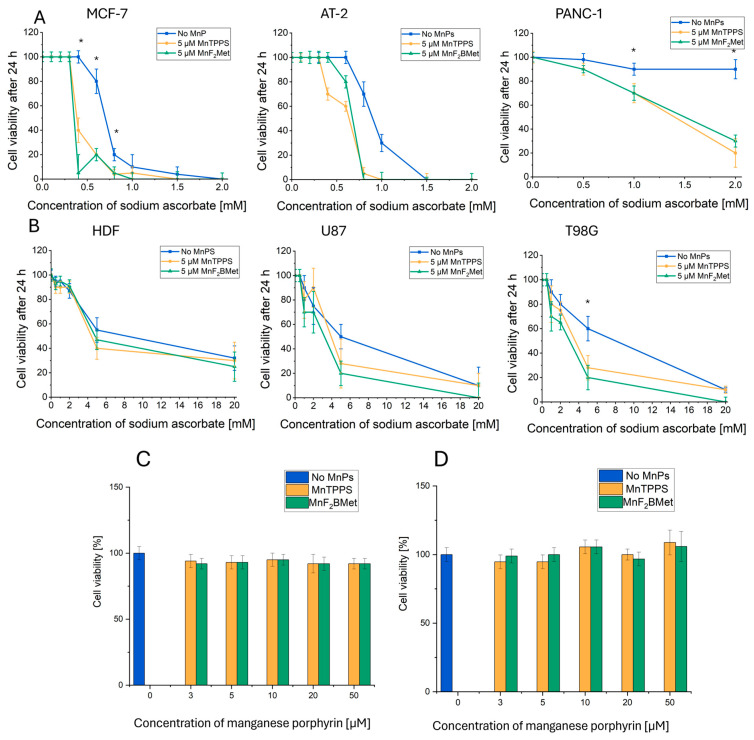
(**A**) Viability of PANC-1, AT-2, and MCF-7 cells assessed using the trypan blue exclusion assay following treatment with 5 μM MnTPPS or 5 μM MnF_2_BMet in the presence of ASC at concentrations ranging from 0 to 2 mM, measured after 24 h. (**B**) Viability of HDF, U87, and T98G cells determined using the trypan blue exclusion assay after treatment with 5 μM MnTPPS or 5 μM MnF_2_BMet in the presence of high concentrations of ASC (0–20 mM), measured after 24 h. Data are presented as mean ± SEM, with viable cells expressed as a percentage of the total cell population, *n* = 3 (* *p* < 0.05). Statistical significance was assessed relative to the control without MnPs. Viability of (**C**) AT-2 cells and (**D**) T98G cells, assessed using the trypan blue exclusion assay following treatment with MnTPPS or MnF_2_BMet over a concentration range of 0 to 50 µM, measured after 24 h. Data are normalized to a DMSO control (0–1%) and presented as mean ± SEM, with viable cells expressed as a percentage of the total cell population.

Among the tested cell lines, MCF-7, AT-2, and PANC-1 exhibited the highest sensitivity to ASC, and viability was substantially reduced upon co-treatment with manganese porphyrins (Figure 2A). In contrast, T98G glioblastoma cells displayed greater resistance to ASC treatment; however, adding MnTPPS or MnF_2_BMet significantly enhanced ASC-induced cytotoxicity (Figure 2B). Interestingly, U87 glioblastoma cells exhibited a response profile similar to that of T98G cells in terms of ASC resistance, but in this case, the presence of manganese porphyrin did not significantly enhance the cytotoxicity of ASC. For both U87 and T98G cells, the survival curves were extended to a broader ASC concentration range (up to 20 mM) to capture differential effects at higher doses. Notably, normal HDFs were the most resistant to ASC treatment, and neither MnTPPS nor MnF_2_BMet enhanced ASC cytotoxicity in these cells (Figure 2B). These results suggest that, under the tested conditions, MnPs can potentiate ascorbate-induced cytotoxicity in several cancer cell lines, with minimal effects observed in normal fibroblasts. This apparent cancer-selective enhancement justified further evaluation of manganese porphyrins as modulators of ascorbate-based strategies. Based on these findings, two cell lines differing in their ASC sensitivity—AT-2 (ASC-sensitive) and T98G (ASC-resistant)—were selected to further investigate the MnPs’ activity at higher concentrations. However, neither MnTPPS nor MnF_2_BMet exhibited direct cytotoxic effects when administered alone at concentrations ranging from 0 to 50 µM (Figure 2C,D).

For subsequent studies, MCF-7 cells were selected as the primary model due to their frequent use in oxidative-stress-based therapeutic strategies involving porphyrins and their well-documented resistance mechanisms [24]. MCF-7 cells are known to overexpress the breast cancer resistance protein (BCRP), which actively effluxes macrocyclic compounds such as pheophorbide a, protoporphyrin IX (PpIX), chlorin e6, and porphyrins, making BCRP-positive tumors challenging targets for porphyrin-based treatments [34,35]. Investigating the MnPs-ASC system in this cell line provides valuable insights into its potential efficacy against resistant breast cancer models.

### 3.3. Effect of MnPs-ASC Treatment on MCF-7 Cell Viability, Proliferation, and Migration

**Figure 3:**
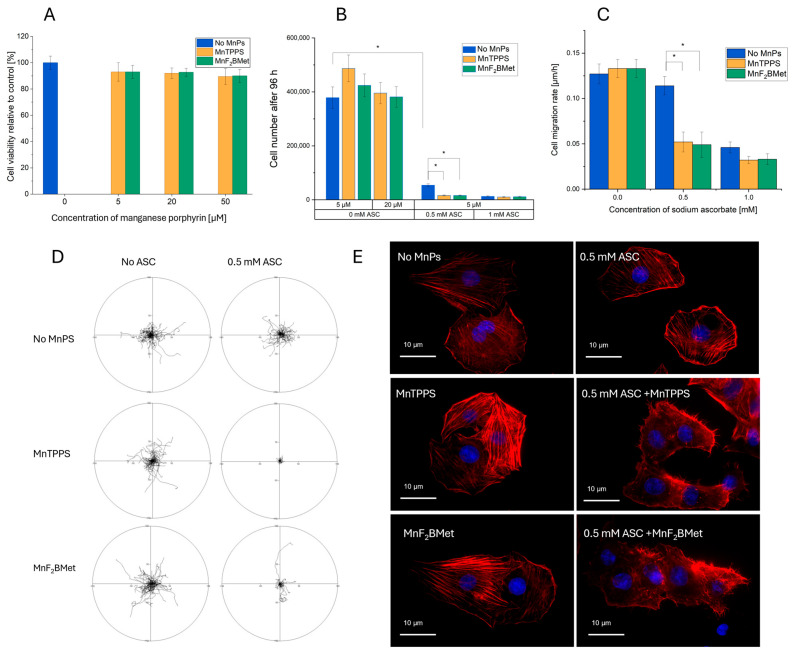
(**A**) Viability of MCF-7 cells, assessed using the trypan blue exclusion assay, following treatment with MnTPPS (5, 20 or 50 μM) or MnF_2_BMet (5, 20 or 50 μM) for 24 h. Data represent mean ± SEM of viable cells as a percentage of total cells, *n* = 3. (**B**) Cell count measured 96 h after treatment with 0.5 or 1 mM ASC in the presence of either 5 or 20 μM MnTPPS or MnF_2_BMet. Data represent mean ± SEM, *n* = 3, * *p* < 0.05. (**C**) Cell migration rate of ASC-treated MCF-7 cells, assessed after ASC exposure. Data represent mean ± SEM of analyzed cells, *n* = 60 (*p* < 0.05). (**D**) Cell movement trajectories determined over an 8 h time-lapse experiment. (**E**) Cell morphology visualized after treatment with 0.5 mM ASC for 6 h, with or without preloading with 5 μM MnTPPS. Red: F-actin; blue: DNA.

To further explore the cytotoxic effects of the MnPs-ASC system on MCF-7 cells, a series of assays were conducted to evaluate cell viability, proliferation, migration, and morphological changes (Figure 3). The viability of MCF-7 cells was assessed using the trypan blue exclusion assay following treatment with 5, 20 or 50 μM MnTPPS or MnF_2_BMet for 24 h (Figure 3A). Neither porphyrin alone significantly affected cell survival. However, when cells were preloaded with 5 μM MnTPPS or 5 μM MnF_2_BMet and subsequently treated with ASC (0–1.5 mM) for 24 h, viability was markedly reduced (Figure 2A). To assess the long-term effects of ASC treatment in the presence of manganese porphyrins, the total number of viable cells was quantified 96 h after treatment (Figure 3B). Co-administration of ASC (0.5 or 1 mM) with 5 or 20 μM MnTPPS or MnF_2_BMet significantly reduced the proliferative capacity of MCF-7 cells compared to those treated with ASC alone, indicating a sustained cytostatic effect. The impact of ASC treatment on cell motility was analyzed by tracking migration rates (Figure 3C) and migration patterns (Figure 3D) after ASC exposure. Cells pretreated with MnPs and subsequently exposed to ASC exhibited a notable reduction in motility, further confirming the inhibitory effects of the MnP-ASC system. Finally, morphological analysis of MCF-7 cells treated with 0.5 mM ASC for 6 h, with or without prior exposure to 5 μM MnTPPS, revealed significant cytoskeletal rearrangements (Figure 3E). Fluorescence imaging demonstrated pronounced actin filament reorganization (red: F-actin) and nuclear condensation (blue: DNA) in MnPs-ASC-treated cells, indicating stress-induced cytoskeletal remodeling. These findings further support the potentiating effect of MnPs on ASC-mediated cytotoxicity in breast cancer cells and underscore the potential of this system as a targeted therapeutic strategy for resistant cancer models.

### 3.4. Kinetics of MCF-7 Cell Death Induced by MnPs-ASC Treatment

**Figure 4:**
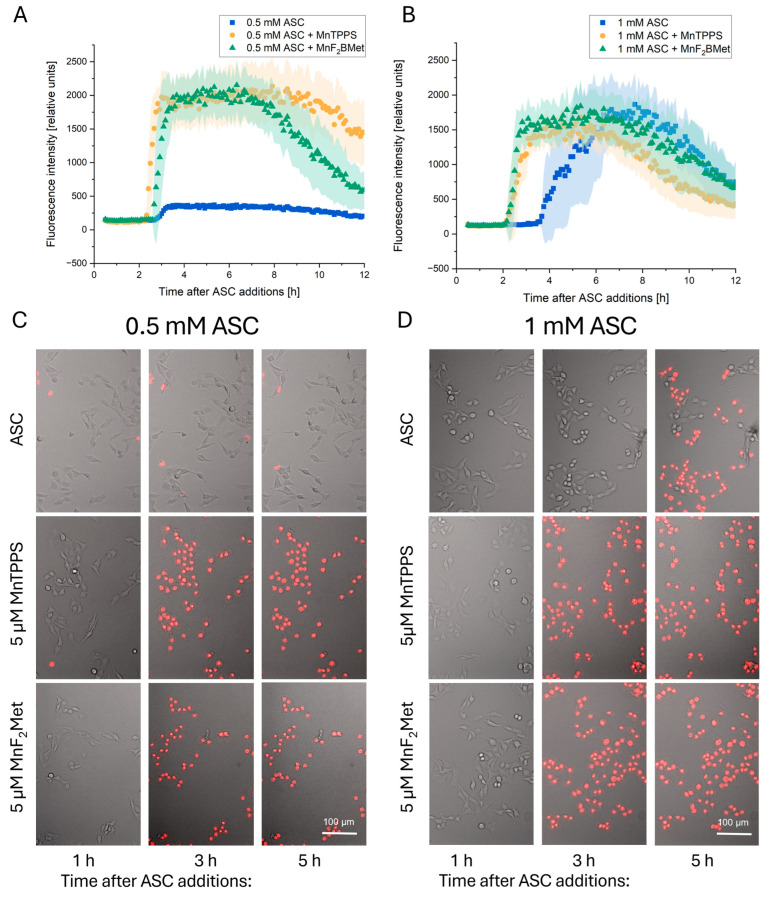
Time-dependent propidium iodide (PI) uptake following ASC administration. PI uptake was monitored as an indicator of loss of membrane integrity in MCF-7 cells preloaded with 5 µM MnTPPS or 5 µM MnF_2_BMet and subsequently treated with 0.5 mM ASC (**A**) or 1 mM ASC (**B**). Data represent mean ± SEM (shaded area) (*n* = 30) In the absence of MnPs, PI uptake was detected 4 h after treatment with 1 mM ASC. However, the presence of manganese porphyrins significantly accelerated this process, leading to detectable PI uptake within 2.5 h after ASC administration. The decrease in fluorescence intensity after about 6 h of imaging is related to cell detachment. Microscopic images of MCF-7 cells captured at 1, 3, and 5 h after treatment with 0.5 mM ASC (**C**) or 1 mM ASC (**D**) illustrate the progressive loss of membrane integrity. Images are composites of integrated modulation contrast (IMC) microscopy and the red fluorescence channel, highlighting PI-positive cells undergoing membrane permeabilization. The scale is applicable to each subgraph.

The observed synergy between manganese porphyrins (MnPs) and ASC in inducing cytotoxic effects prompted an investigation into the kinetics of this process. To determine the timing and progression of ASC-induced MCF-7 cell death in the absence and presence of MnPs, propidium iodide (PI) uptake was monitored using time-lapse video microscopy as an indicator of membrane integrity loss (Figure 4).

When applied alone, MnTPPS and MnF_2_BMet did not induce PI uptake during the first 12 h following drug administration, confirming their lack of intrinsic cytotoxicity. Similarly, ASC at 0.5 mM did not exhibit direct toxicity and failed to induce PI uptake in MCF-7 cells. However, when 5 µM MnTPPS or 5 µM MnF_2_BMet was introduced prior to ASC exposure, a marked cytotoxic response was observed, with PI uptake occurring within 2 to 4 h, depending on the ASC concentration (Figure 4A,C; Videos S1, S2, S3). At 1 mM, ASC alone was sufficient to induce cytotoxicity, leading to cell death approximately 4 h after treatment. Notably, the presence of MnTPPS or MnF_2_BMet significantly accelerated this process, reducing the time required for detectable PI uptake to 2 h (Figure 4B,D; Videos S4, S5, S6). These results indicate that MnPs enhance ASC’s cytotoxic properties by accelerating the loss of membrane integrity, ultimately leading to the rapid and effective induction of cell death in MCF-7 cells.

### 3.5. Hallmarks of Oxidative Stress Induced by the MnPs-ASC System

**Figure 5:**
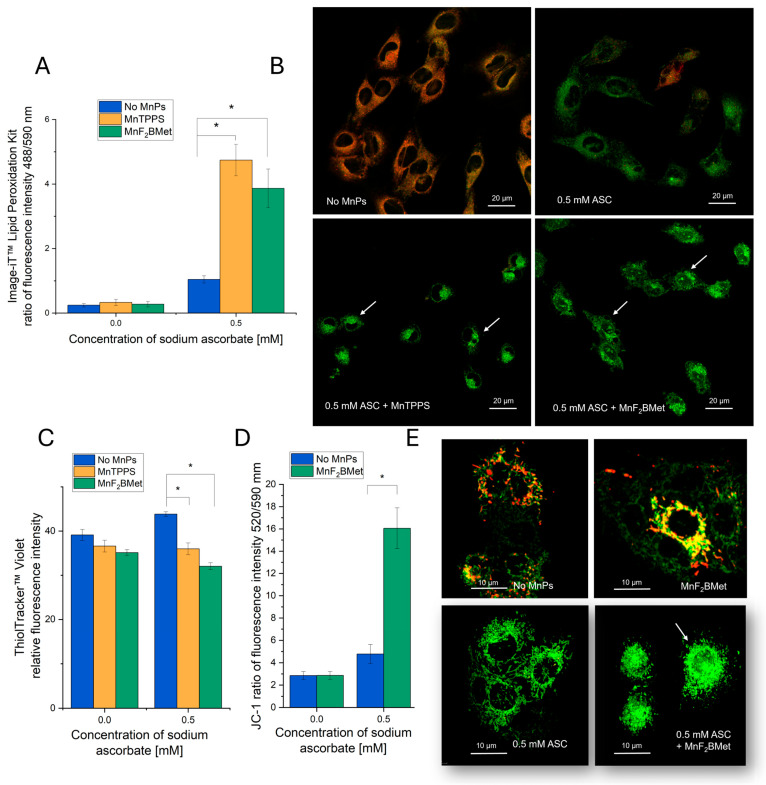
Manganese porphyrins combined with ASC induce oxidative cellular damage. (**A**) Lipid peroxidation in MCF-7 cells, assessed 2 h after ASC administration using the Image-iT™ Lipid Peroxidation Kit. (**B**) Representative fluorescence images show merged channels (red—no peroxidation; green—peroxidase lipid), with arrows indicating morphological changes associated with oxidative stress. (**C**) Intracellular glutathione (GSH) levels, measured 2 h after exposure to 0.5 mM ASC in MCF-7 cells preloaded with 5 µM MnTPPS or 5 µM MnF_2_BMet, using ThiolTracker™ Violet assay and fluorescence microscopy. (**D**) Mitochondrial membrane potential, assessed using the JC-1 assay. MCF-7 cells loaded with 5 µM MnF_2_BMet and treated with 0.5 mM ASC. (**E**) Representative fluorescence images highlight mitochondrial morphology alterations, with arrows indicating regions of structural disruption (red—high mitochondrial membrane potential; green—low mitochondrial membrane potential). Data represent mean ± SEM (*n* = 50, * *p* < 0.05).

To further elucidate the mechanisms underlying early cell membrane damage induced by the MnPs-ASC system in MCF-7 cells, we investigated key hallmarks of oxidative stress following MnPs and/or ASC treatment. These analyses revealed that the membrane integrity loss observed in the previous experiments (Figure 4) is accompanied by a significant increase in lipid peroxidation in cells treated with 0.5 mM ASC in the presence of 5 µM MnTPPS or 5 µM MnF_2_BMet. While 0.5 mM ASC alone also induced lipid peroxidation within 2 h after treatment, this effect was less pronounced compared to what was observed for cells preloaded with MnPs (Figure 5A,B).

In parallel with the observed lipid peroxidation, we detected a substantial depletion of intracellular glutathione (GSH) and mitochondrial membrane depolarization in MnPs-ASC-treated MCF-7 cells. A statistically significant decrease in GSH levels was observed upon ASC administration in cells preloaded with 5 µM MnTPPS or 5 µM MnF_2_BMet (Figure 5C). Notably, MnPs alone did not alter intracellular GSH levels in these conditions. Interestingly, treatment with 0.5 mM ASC alone led to a moderate increase in the intracellular GSH pool, which may reflect a compensatory cellular response to oxidative stress. Finally, we observed that MnPs-ASC treatment also caused mitochondrial membrane depolarization, which was significantly more pronounced in the cells preloaded with 20 µM MnF_2_BMet. These alterations were accompanied by changes in mitochondrial structure, including mitochondrial fragmentation and loss of network continuity, as visualized by fluorescence microscopy (Figure 5D,E).

Caspase-3/7 activation was detected between 2 and 3 h after treatment with 0.5 mM ASC in cells loaded with MnTPPS or MnF2BMet. No activation was observed in untreated cells, cells exposed to MnPs alone, or cells treated with 0.5 mM ASC in the absence of MnPs. In cells treated with 1 mM ASC, an increase in the Caspase-3/7 Detection Reagent signal was observed regardless of MnPs cell loading (Figure S1).

Collectively, these findings indicate that MnPs enhance ASC-induced oxidative stress, leading to membrane lipid peroxidation, depletion of intracellular antioxidant defenses, and mitochondrial dysfunction, ultimately contributing to cellular damage and death.

### 3.6. Combined MnPs/ASC Treatment Increases H_2_O_2_ Levels in MCF-7 Cells

**Figure 6:**
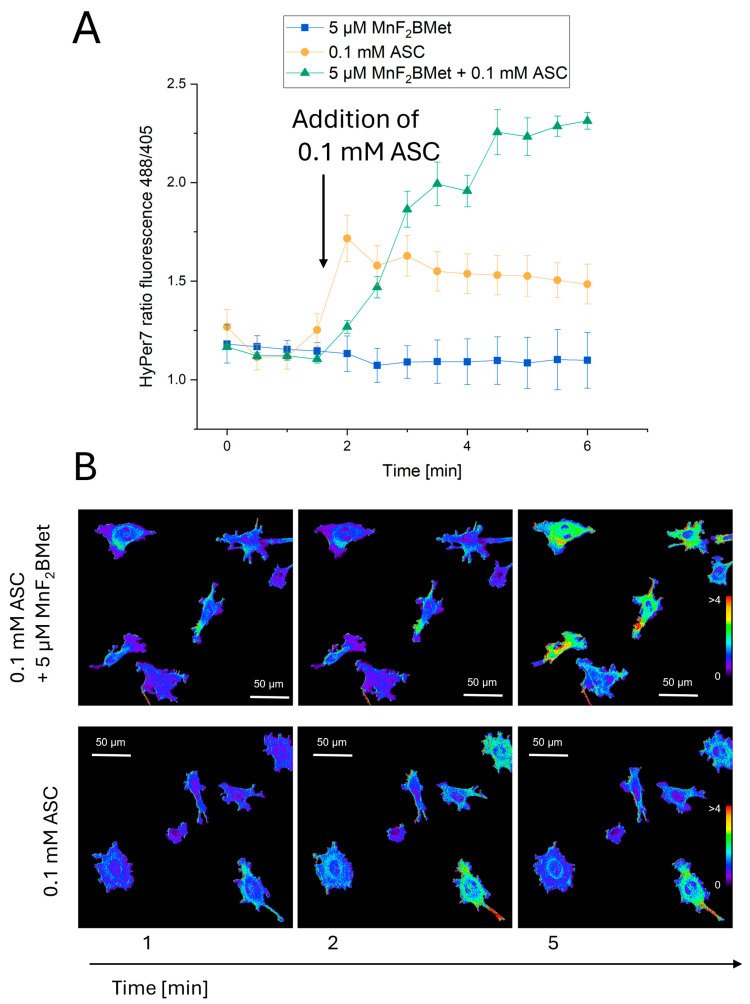
Detection of intracellular hydrogen peroxide (H_2_O_2_) using the HyPer7 biosensor. (**A**) MCF-7 cells preloaded with 5 µM MnF_2_BMet were illuminated every 30 s with 405 nm and 488 nm lasers over the course of a 6 min experiment to establish a baseline fluorescence level and confirm that MnF_2_BMet does not generate intracellular H_2_O_2_ upon light exposure. ASC was added after 90 s. In the absence of MnPs, the addition of 0.1 mM ASC caused a rapid increase in H_2_O_2_ levels, followed by a gradual decline in fluorescence intensity. In contrast, when 0.1 mM ASC was added to cells preloaded with 5 µM MnF_2_BMet, the increase in H_2_O_2_ levels was slower but reached a maximum that was 2.5 times higher than in ASC-treated cells without MnPs. Data represent mean ± SEM, *n* = 30. (**B**) Fluorescence microscopy images of MCF-7 cells captured at 1, 2, and 5 min of live-cell imaging, either in the presence of 5 µM MnF_2_BMet or in the absence of MnPs (control cells). ASC was introduced between the 1st and 2nd minutes of live-cell imaging. The images represent ratiometric data, with the fluorescence signal excited at 488 nm divided by the signal excited at 405 nm. The pseudocolor scale reflects the fluorescence ratio.

To further elucidate the mechanisms underlying oxidative stress induction in MnPs/ASC-treated MCF-7 cells, we investigated intracellular H_2_O_2_ levels using the HyPer7 biosensor. As a control, MCF-7 cells preloaded with 5 µM MnF_2_BMet were repeatedly illuminated for 6 min, with no detectable changes in H_2_O_2_ levels or cellular morphology (Figure 6), both confirmed with a subsequent 27 min of imaging (Figure S2). These findings confirm that 5 µM MnF_2_BMet alone does not contribute to H_2_O_2_ generation under these experimental conditions and does not interfere with the measurement when ASC is added. This is particularly relevant given that the free porphyrin ligand is photochemically active and able to generate ROS. The lack of ROS production in the presence of Mn(III)-complexed porphyrin indicates that no free ligand molecules remain in the system, thereby confirming the full coordination of manganese.

In contrast, introducing 0.1 mM ASC into the culture medium resulted in a rapid and pronounced increase in the H_2_O_2_-specific signal, reaching a maximum intensity within 30 s, followed by a gradual decline (Videos S7, S8). Notably, when ASC was added to cells preloaded with 5 µM MnF_2_BMet, the increase in H_2_O_2_ levels was significantly higher than in ASC-treated cells without porphyrin pretreatment （Videos S9, S10).

These results confirm that the light-induced enhancement of the HyPer7 fluorescence signal likely reflects localized ROS amplification, potentially involving photoactivation of membrane-bound MnPs or intracellular processes facilitated by ASC-induced redox cycling. Although HyPer7 is considered a highly selective probe for H_2_O_2_, the contribution of other ROS cannot be entirely excluded, particularly under conditions involving light exposure and redox-active metal complexes. Thus, photo-assisted redox processes have been proposed in related systems [36]; while HyPer7 is selective for H_2_O_2_, secondary ROS could contribute to membrane damage. Our findings point to H_2_O_2_ as the principal ROS involved, but they also suggest that, under light exposure, a broader ROS network may be activated, further amplifying the cytotoxic effects of the MnPs–ASC system.

### 3.7. Extracellular Interactions Between ASC and MnPs Mediate Their Combined Cytotoxicity

**Figure 7:**
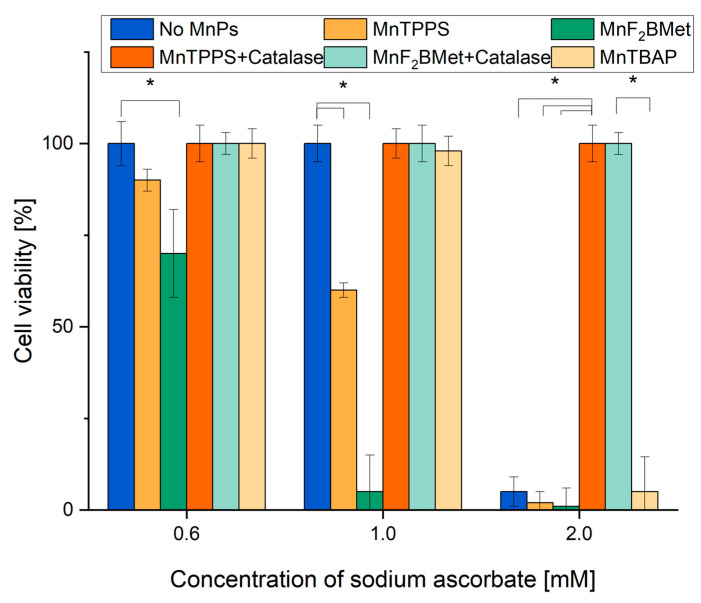
MCF-7 cell viability following MnPs-ASC treatment and the effect of extracellular catalase. Viability of MCF-7 cells was assessed using the trypan blue exclusion assay after 24 h of treatment with 5 µM MnTPPS or 5 µM MnF_2_BMet in the presence of ASC (0.6, 1, and 2 mM). Data are expressed as mean ± SEM, (* *p* < 0.05), with viable cells presented as a percentage of the total cell population.

Given that both ASC and MnPs are present in the extracellular space and can also accumulate intracellularly, the H_2_O_2_ generation induced by MnPs/ASC interactions may occur in both compartments [22]. To determine whether extracellular or intracellular ROS production is responsible for the cytotoxic effects of the MnPs-ASC system, we examined the impact of exogenously added catalase on MCF-7 cell viability (Figure 7). As an additional control, we included MnTBAP, used here as a reference manganese porphyrin with low catalytic activity toward ascorbate [37]. Our results show that extracellular catalase completely abolished the cytotoxic effects of the MnPs-ASC system, indicating a predominant contribution of extracellular H_2_O_2_ to the observed response. This conclusion was further reinforced by experiments utilizing 1 mM DHA, a non-radical form of ASC that is taken up by cells via GLUTs and subsequently reduced to ASC intracellularly. While DHA alone remained non-toxic to MCF-7 cells, its addition to 5 µM MnF_2_BMet-loaded cells resulted in only a minor, statistically insignificant decrease in viability, supporting the notion that intracellular ASC alone is insufficient to drive the MnPs-mediated cytotoxic response.

**Figure 8:**
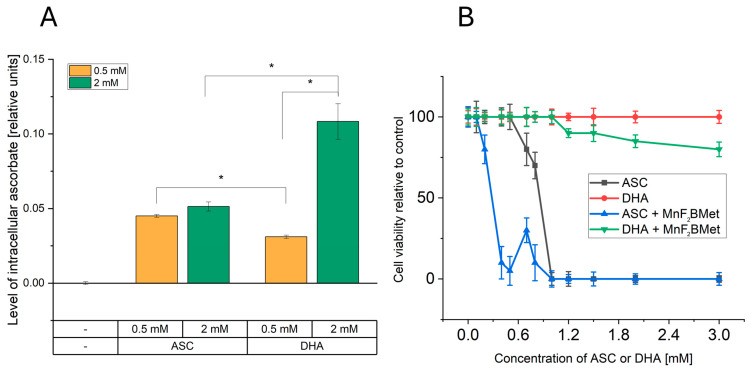
DHA enhances intracellular ASC levels but exhibits limited cytotoxicity in MCF-7 cells. (**A**) Intracellular ASC levels following the administration of 0.5 or 2 mM ASC or 0.5 or 2 mM DHA in MCF-7 cells. While 0.5 and 2 mM ASC resulted in a similar increase in intracellular ASC levels, 0.5 mM DHA was less effective in delivering ASC to cancer cells compared to its reduced form. The most efficient method for increasing intracellular ASC levels was the administration of 2 mM DHA, indicating a concentration-dependent effect, (* *p* < 0.05). (**B**) Cytotoxic effects of ASC and DHA on MCF-7 cells. Unlike ASC, DHA alone did not exhibit cytotoxic properties in MCF-7 cells. However, the presence of 5 µM MnF_2_BMet led to a moderate decrease in cell viability (~80%) after 24 h of DHA treatment. In contrast, under the same conditions, ASC treatment alone and ASC combined with 5 µM MnF_2_BMet resulted in a significant reduction in cell viability, further supporting the role of ASC in MnPs-induced cytotoxicity.

Importantly, a significant increase in intracellular ASC levels was observed 6 h after adding 2 mM DHA (Figure 8). However, despite the intracellular accumulation of ASC, no substantial cytotoxic effects were observed, supporting the view that extracellular ascorbate is critical for the MnPs-ascorbate response under our conditions. Consistent with this mechanism, extracellular catalase fully neutralized the cytotoxic effects of MnPs-ASC treatment (Figure 7), underscoring the central role of H_2_O_2_ production in mediating cell death. Additionally, MnTBAP (5 µM) did not enhance ASC cytotoxicity, further demonstrating that the redox activity of MnTPPS and MnF_2_BMet is essential for their potentiating effects. These findings collectively support extracellular ascorbate oxidation as the main contributor to MnPs-ascorbate cytotoxicity, whereas intracellular ascorbate accumulation alone was insufficient in our assays.

## 4. Discussion

The hypothesis that manganese porphyrins catalyze ascorbate oxidation, yielding ascorbyl radicals and hydrogen peroxide (H_2_O_2_) with preferential toxicity toward cancer cells, has been widely investigated [21,22,24,37,38]. MnPs are recognized as potent redox-active catalysts that facilitate the formation of ROS from ASC, which preferentially damages cancer cells due to their heightened sensitivity to oxidative stress and impaired antioxidant defenses [3]. Despite the promising results of previous studies, the precise mechanisms underlying MnP-ASC cytotoxicity remain only partially understood. Notably, in several studies, the major mediating role of H_2_O_2_ was inferred from indirect markers such as GSH depletion or lipid peroxidation, with fewer reports providing direct H_2_O_2_ readouts [39]. Furthermore, both intracellular and extracellular sources have been discussed, although multiple studies have supported a predominant extracellular contribution under specific conditions [21,38,39].

Here, we evaluated two synthetic MnPs (MnTPPS and MnF_2_BMet) for their ability to efficiently catalyze ASC oxidation, generate H_2_O_2_, and exert cytotoxic effects in cancer cells. Our study indicates a predominant contribution of extracellular H_2_O_2_ to the observed cytotoxicity, reinforcing the relevance of extracellular ROS dynamics in MnP–ascorbate responses.

Previous studies have reported that some MnPs exhibit intrinsic cytotoxicity when administered alone [40]. However, our results demonstrate that MnTPPS and MnF_2_BMet, when applied at standard in vitro concentrations (5–20 µM), do not induce significant cytotoxicity, nor do they affect the migratory or proliferative potential of cancer cells [21,24,40]. These findings align with earlier observations regarding the redox properties of manganese porphyrins in breast cancer models [22,40,41,42]. In contrast, when combined with ASC, both MnPs significantly enhance ASC-induced cytotoxicity in MCF-7 cells, consistent with the proposed mechanism of ASC oxidation and subsequent ROS generation. The extent of ascorbate oxidation by manganese porphyrins, resulting in H_2_O_2_ generation, depends on their redox and structural properties [24]. To date, numerous MnPs have been developed that catalyze ASC oxidation, generating ROS such as superoxide (O_2_^•−^) and H_2_O_2_. Among these, 5″N-substituted pyridyl-porphyrins have been specifically designed as superoxide dismutase (SOD) mimetics [40]. Notably, the ASC concentrations used in our experiments are physiologically relevant, corresponding to plasma levels observed in patients receiving pharmacological ASC administration [11,20]. Some studies have even reported ASC concentrations as high as 20 mM in patient sera shortly after intravenous administration [15]. Thus, our findings add to reports of ROS-dependent cytotoxicity in cancer models and support further evaluation of this approach.

To date, most studies on the prooxidative activity of MnPs have been based on oxygen consumption assays and indirect detection of H_2_O_2_ in cell-free systems [37]. Numerous MnPs have been shown to generate ROS under cell-free conditions, particularly when combined with high concentrations of ASC [21,22,38]. These findings indirectly suggest that oxidative stress is induced in cells treated with MnPs/ASC; however, the intracellular environment is highly complex and contains many redox-active species that can interact with MnPs, including thiols, superoxide anions (O_2_^•−^), and hydrogen peroxide. Moreover, local pH fluctuations further modulate these interactions [21]. Previous in vitro studies have suggested that MnP-ASC interactions lead to ROS generation, but these conclusions were mostly based on indirect markers such as GSH depletion or lipid peroxidation. It has been demonstrated that H_2_O_2_ is crucial for the cytotoxic properties of MnP-ASC, as the application of extracellular catalase abolished the cytotoxic effect. This indicates an extracellular mechanism of ROS generation.

In this study, we directly quantified intracellular H_2_O_2_ levels using the HyPer7 fluorescent probe, providing clear evidence that H_2_O_2_ is the key mediator of MnP-ASC-induced oxidative stress in MCF-7 cells. This method allowed us to demonstrate that MnP-ASC treatment leads to sustained intracellular H_2_O_2_ elevation, correlating with increased lipid peroxidation, mitochondrial membrane depolarization, and ultimately cell death. The trypan blue exclusion test and PI uptake are complementary assays that indicate the loss of cell membrane integrity, but they cannot detect cells in the early stages of apoptosis. On the other hand, the PI uptake kinetics are consistent with rapid membrane integrity loss; however, definitive discrimination between necrosis and apoptosis would require dedicated markers [13,24,43,44,45]. The experiments demonstrated that caspases 3 and 7 were activated upon treatment with the MnP–ASC system (Figure S1); however, the timing of cell membrane damage is too rapid for the finalization of apoptosis. These findings indicate a complex mechanism of cell death in which apoptotic pathways may be initiated, but excessive ROS accumulation leads to membrane damage and uncontrolled cell death. We also do not exclude the possibility of ferroptosis associated with lipid peroxidation, which requires further investigation to clarify the underlying cell death mechanism. Our results support extracellular H_2_O_2_ as a main contributor to MnP–ascorbate cytotoxicity under our conditions. Extracellular catalase completely abolished MnP-ASC cytotoxic effects, confirming that H_2_O_2_ is generated outside the cells and must diffuse intracellularly to exert its effects. DHA supplementation (which enters cells and is reduced to ASC intracellularly) failed to induce significant cytotoxicity, even though it increased intracellular ASC levels.

Our observations with DHA further support the conclusions drawn from the catalase experiments, namely, that ROS generation occurs predominantly extracellularly. This is because DHA supplementation increases ascorbate levels only inside the cells—DHA is reduced to ascorbate exclusively intracellularly—and, consistently with this, DHA failed to induce cytotoxicity [46]. At first glance, this may appear inconsistent with our experimental design, in which MnPs are washed out prior to ascorbate addition, seemingly suggesting an intracellular mechanism driven by MnPs that have already entered the cells. However, we cannot exclude that a fraction of MnPs become adsorbed to the cell surface or are actively exported from the intracellular space, allowing these MnPs to remain accessible extracellularly even after washing. Thus, we propose that H_2_O_2_ is generated outside the cells by MnPs retained at or returned to the extracellular surface, rather than by fully internalized MnPs.

Although the manganese porphyrins used in this study do not fluoresce and therefore cannot be tracked by conventional fluorescence microscopy, several observations indicate that at least a fraction of these compounds may remain associated with the plasma membrane. The rapid onset of membrane damage, the full cytoprotection by extracellular catalase, and the inability of intracellularly generated ascorbate (via DHA uptake) to induce comparable toxicity all support the view that ROS formation occurs close to the cell surface. This interpretation is also consistent with our previous work on structurally related porphyrins. In a comparative study of F_2_PMet and ZnF_2_PMet, we demonstrated that zinc coordination increases interaction with membrane proteins and leads to a more diffuse, membrane-associated distribution, whereas the free-base porphyrin localizes predominantly in the ER and mitochondria [47]. Although Mn(III) porphyrins cannot be visualized directly, their structural similarity to these compounds suggests that a modest but functionally significant membrane association is plausible and may permit catalysis of extracellular ascorbate oxidation even after washing steps.

Collectively, these findings confirm the role of extracellular interactions between manganese porphyrins and ascorbate in H_2_O_2_ formation at or near the cell surface. A similar mechanism has been described in the degradation of hyaluronic acid, which depends on MnP-ASC interactions in the extracellular milieu [39]. Based on these observations, we propose a mechanistic model in which (i) MnPs accumulate on the cancer cell surface, possibly through electrostatic interactions with extracellular proteins or altered glycocalyx structures; (ii) in the presence of ASC, MnPs catalyze the extracellular accumulation of H_2_O_2_ and its diffusion into the cells; (iii) locally accumulated ROS lead to cell membrane damage, whereas intracellular H_2_O_2_ overwhelms antioxidant defenses (GSH depletion), induces lipid peroxidation, and disrupts mitochondrial function, leading to (iv) irreversible damage and necrotic cell death. These findings support an extracellular ROS-linked mechanism of cancer cell death and lay the foundation for further studies to define conditions under which such redox modulation could be therapeutically leveraged.

## 5. Conclusions

Our results indicate that MnTPPS and MnF_2_BMet can enhance the cytotoxic effects of ascorbate, consistent with a mechanism in which extracellular hydrogen peroxide (H_2_O_2_) generation plays a predominant role. The resulting H_2_O_2_ likely diffuses into cells, where it causes oxidative damage to organelles and membranes, leading to loss of cell integrity, while sparing normal fibroblasts under the tested conditions. These findings support the potential of manganese porphyrin–ascorbate redox systems, in which a metal-based catalyst promotes ascorbate oxidation and the formation of reactive peroxide species. Further development of this concept should focus on (i) optimizing the manganese porphyrin structure to improve redox efficiency and selectivity; (ii) evaluating the therapeutic relevance of MnP–ascorbate interactions in vivo in tumors with altered redox balance; (iii) assessing interactions between MnPs, ascorbate, and extracellular matrix components that may modulate their localization and ROS generation; and (iv) combining MnP–ascorbate treatment with other oxidative-stress-based modalities, such as photodynamic therapy, radiotherapy, or chemotherapy. By refining these approaches, MnP–ascorbate systems may evolve into a promising platform for redox-based cancer therapy that selectively exploits the oxidative vulnerabilities of tumor cells.

## Data Availability

The datasets used and/or analyzed in this study are available from the corresponding author upon reasonable request.

## Supplementary Materials

The following supporting information can be downloaded at: https://www.mdpi.com/article/10.3390/cancers17233736/s1. Video S1: Treatment with 0.5 mM ASC. PI uptake in MCF-7 cells over time. Video starts 30 min after ASC addition. Video S2: Treatment with 0.5 mM ASC + 5 µM MnTPPS. PI uptake in MCF-7 cells over time. Video starts 30 min after ASC addition. Video S3: Treatment with 0.5 mM ASC+ 5 µM MnF_2_BMet. PI uptake in MCF-7 cells over time. Video starts 30 min after ASC addition. Video S4: Treatment with 1 mM ASC. PI uptake in MCF-7 cells over time. Video starts 30 min after ASC addition. Video S5: Treatment with 1 mM ASC + 5 µM MnTPPS. PI uptake in MCF-7 cells over time. Video starts 30 min after ASC addition. Video S6: Treatment with 1 mM ASC + 5 µM MnF_2_BMet. PI uptake in MCF-7 cells over time. Video starts 30 min after ASC addition. Video S7: HyPer7 fluorescence ratio over time. Treatment with 0.1 mM ASC. ASC addition after 90 s of live-cell imaging. The grayscale corresponds to the ratio of fluorescence intensity: black—fluorescence ratio = 0; white—fluorescence ratio > 4. Video S8: HyPer7 fluorescence ratio over time. Treatment with 0.1 mM ASC. ASC addition after 90 s of live-cell imaging. The color scale corresponds to the ratio of fluorescence intensity: black—fluorescence ratio = 0; red—fluorescence ratio > 4. Video S9: HyPer7 fluorescence ratio over time. Treatment with 0.1 mM ASC + 5 µM MnF_2_BMet. ASC addition after 90 s of live-cell imaging. The grayscale corresponds to the ratio of fluorescence intensity: black—fluorescence ratio = 0; white—fluorescence ratio > 4. Video S10: HyPer7 fluorescence ratio over time. Treatment with 0.1 mM ASC + 5 µM MnF_2_BMet. ASC addition after 90 s of live-cell imaging. The color scale corresponds to the ratio of fluorescence intensity: black—fluorescence ratio = 0; red—fluorescence ratio > 4. Figure S1: Activation of caspase-3/7 induced by the MnP–ASC system. The images show the signal from the CellEvent™ Caspase-3/7 Detection Reagents (green) overlaid on bright-field microscopy images of the cells. Figure S2: Control imaging of cells loaded with the HyPer7 probe for up to 7 min. Additionally, control imaging of cells loaded with 5 µM MnF2BMet and the HyPer7 probe for up to 27 min under experimental conditions. Data represent mean ± SEM, *n* = 7.

## Abbreviations

The following abbreviations are used in this manuscript:

MnPs	Manganese porphyrins
ASC	Ascorbate
HDF	Human dermal fibroblasts
DHA	Dehydroascorbate
PDT	Photodynamic therapy
ROS	Reactive oxygen species
AA	L-ascorbic acid
SOD	Superoxide dismutase
GSH	Glutathione
MnTBAP	Mn(III) tetrakis(4-benzoic acid) porphyrin chloride
MnTPPS	Manganese(III) porphyrin: 5,10,15,20-Tetrakis(4-sulfonylphenyl)porphyrin manganese(III) acetate
MnF_2_BMet	5,10,15,20-Tetrakis [2,6-difluoro-5(N-methylsulfamoyl)phenyl]porphyrin manganese(III)
RPMI 1640	Roswell Park Memorial Institute 1640 medium
DMSO	Dimethyl sulfoxide
FBS	Fetal bovine serum
PBS	Phosphate-buffered saline
