# Supplementary figures and images for: Targeting Cancer with Redox Catalysis: Manganese Porphyrins and Ascorbate Synergistically Induce Selective Oxidative Stress and Necrotic Cell Death
Source: Cancers (Basel). 2025 Nov 22;17(23):3736. doi: 10.3390/cancers17233736 (PMC12691324; doi:10.3390/cancers17233736)

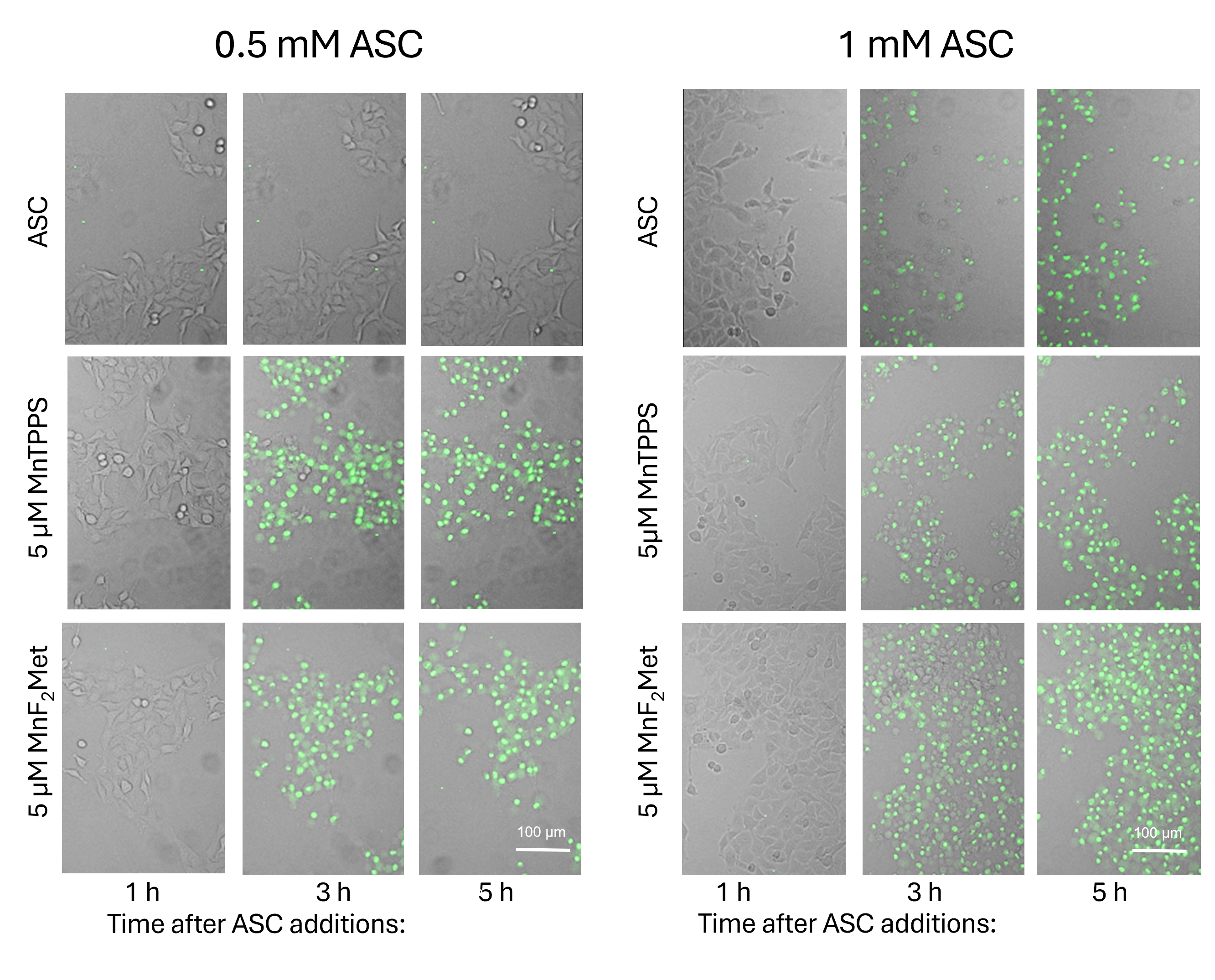

Supplement: Supplementary file 1 [file cancers-17-03736-s001.zip › Figure S1.png]

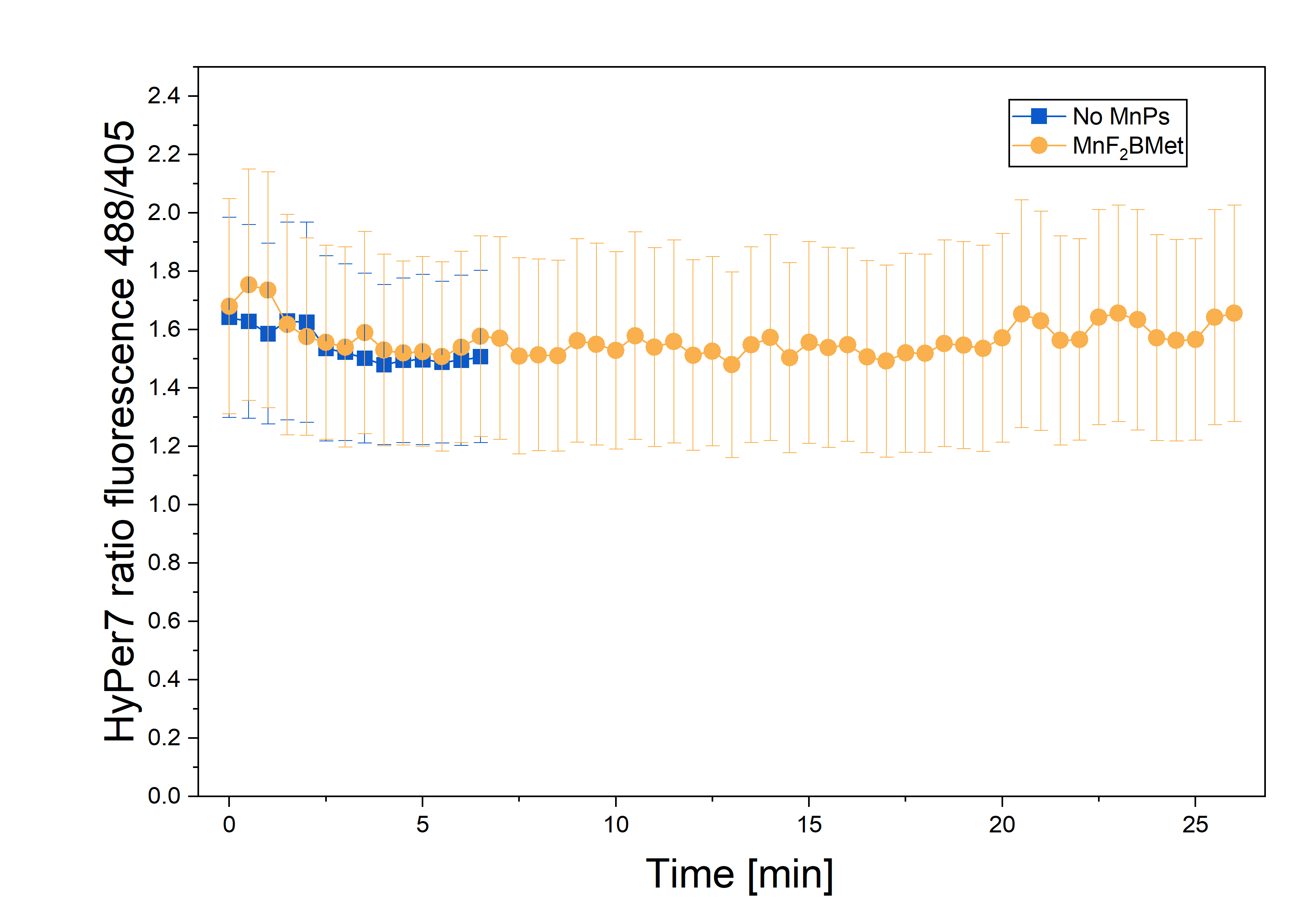

Supplement: Supplementary file 1 [file cancers-17-03736-s001.zip › Figure S2.png]
